# Heterophase-structured nanocrystals as superior supports for Ru-based catalysts in selective hydrogenation of benzene

**DOI:** 10.1038/srep39847

**Published:** 2017-01-06

**Authors:** Zhikun Peng, Xu Liu, Shuaihui Li, Zhongjun Li, Baojun Li, Zhongyi Liu, Shouchang Liu

**Affiliations:** 1 School of Chemistry and Molecular Engineering, Zhengzhou University, 100 Kexue Avenue, Zhengzhou 450001, P R China

## Abstract

ZrO_2_ heterophase structure nanocrystals (HSNCs) were synthesized with tunable ratios of monoclinic ZrO_2_ (*m*-ZrO_2_) to tetragonal ZrO_2_ (*t*-ZrO_2_). The phase mole ratio of *m*-ZrO_2_ versus *t*-ZrO_2_ in ZrO_2_ HSNCs was tuned from 40% to 100%. The concentration of the surface hydroxyl groups on *m*-ZrO_2_ is higher than that on *t*-ZrO_2_. ZrO_2_ HSNCs have different surface hydroxyl groups on two crystalline phases. This creates more intimate synergistic effects than their single-phase counterparts. The ZrO_2_ HSNCs were used as effective supports to fabricate heterophase-structured Ru/ZrO_2_ catalysts for benzene-selective hydrogenation. The excellent catalytic performance including high activity and selectivity is attributed to the heterogeneous strong/weak hydrophilic interface and water layer formed at the *m*-ZrO_2_/*t*-ZrO_2_ catalyst junction.

Heterophase structures, sometimes called heterojunctions^1^,^2^,^3^, have unique physical and chemical properties due to the synergy between various physical properties and overlapping electronic energy levels^4^,^5^,^6^. The junctions of heterophase structures are frequently intriguing sites for physical and chemical processes including photocatalysis^7^,^8^,^9^,^10^,^11^. The heterophase structures benefit the separation and transfer of the electron-hole pairs and the effective utilization of visible light. This is the main reason for their excellent catalytic performances^9^,^10^,^11^. Therefore, heterophase structures effectively improve heterogeneous reactions^12^,^13^.

ZrO_2_ exhibits some advantages as catalyst supports in some reactions due to its amphoteric surface properties and stability under oxidizing and reducing environments^14^,^15^,^16^,^17^,^18^,^19^,^20^,^21^,^22^,^23^,^24^. Under atmospheric pressure, the ZrO_2_ exists in three crystalline phases: *m-*ZrO_2_, *t*-ZrO_2_ and amorphous phase (*am*-ZrO_2_). The phase transformation can be achieved by controlling the synthesis parameters and post-treatment conditions. The surface electronic properties on the acid sites and surface hydroxyl groups of ZrO_2_ can be designed, synthesized and used as supports for heterogeneous catalysts in some important green reactions^15^,^17^,^18^.

Because of the wide use of cyclohexene and its complicated traditional production routes, the benzene-selective hydrogenation is of great industrial importance for affordable and environmentally benign cyclohexene production^25^,^26^. The reaction system is a very complex four-phase system, including two liquid phases, a gas phase, and a solid phase^26^. It is still very challenging to achieve a high selectivity and yield of cyclohexene with high activity from benzene due to the severe thermodynamic limitations. Currently, Ru-based catalysts are the most effective, but they always tend to produce cyclohexane with high activity. Many developments of Ru-based heterogeneous catalysts are trying to solve this long-standing problem^25^,^26^,^27^,^28^,^29^,^30^. Most studies have enhanced the selectivity via tuning and controlling the catalytic active components, co-catalysts, and additives^31^,^32^,^33^,^34^,^35^,^36^. A common view in most researches is that the hydrophilic/hydrophobic properties of the catalysts or supports are of great concern^25^,^26^,^27^,^28^,^29^,^30^,^31^,^32^,^33^,^34^,^35^,^36^. To date, relatively little attention has been given to constructing the heterophase structures of the catalysts and tuning the phase structure of supports for more appropriate surface compatibility^12^.

In this article, ZrO_2_ HSNCs with different phase mole ratios of *m-*ZrO_2_ versus *t-*ZrO_2_ were synthesized from aqueous solutions of ZrOCl_2_·8H_2_O at different conditions. The heterophase-structured Ru/ZrO_2_ catalysts were fabricated using ZrO_2_ HSNCs as supports. The phase mole ratio of ZrO_2_ HSNCs significantly affects the performance of the Ru/ZrO_2_ catalysts in benzene-selective hydrogenation to cyclohexene. We think that a heterogeneous water layer and oil layer formed at the *m*-ZrO_2_/*t*-ZrO_2_ junction of catalysts in the reaction system, and that the specific diffusion-restricted area accounts for the superior catalytic behavior.

## Experimental Section

### Materials

RuCl_3_·3H_2_O (Ru: 37 wt%) was purchased from Sino-platinum Metals CO., LTD, China. Other chemicals including NaOH, NaBH_4_, ZrOCl_2_·8H_2_O, NH_4_HCO_3_, benzene, cyclohexene, and cyclohexane were purchased from the Beijing chemical Co., LTD, China and used without further purification. Deionized water was used in all experiments.

### Synthesis of ZrO_2_ HNCs

ZrO_2_ HSNCs were synthesized according to the following procedures. Aqueous solutions of ZrOCl_2_·8H_2_O and NH_4_HCO_3_ were added to a flask under vigorous agitation using a parallel-flow method. Typically, fixing the mole ratio of n (NH_4_HCO_3_)/n (zirconium ions) at 2 and the pH between 5.1~5.8 ensures that the zirconium ions complete precipitation. The resulting white precipitates were filtrated after aging for 24 h and washed with deionized water until no chlorine was detected. The hydrous precipitates were then transferred into a distillation flask for azeotropic distillation with *n*-butanol. At 93 °C, the azeotropes of water and *n*-butanol were distilled, and the excess *n*-butanol was distilled continuously at 118 °C. Loose white powders of ZrO_2_ precursor were obtained without any residual *n*-butanol. Finally, the remaining powders were calcined at 400, 600, 800, and 1000 °C, respectively, at 10 °C min^−1^ in a muffle for 2 h. Four ZrO_2_ samples with different phase mole ratios of *m*-ZrO_2_ versus *t*-ZrO_2_ were synthesized and denoted as ZrO_2_(A), ZrO_2_(B), ZrO_2_(C), and ZrO_2_(D), respectively.

### Catalyst preparation

The Ru/ZrO_2_ catalysts were prepared using the four ZrO_2_ samples as supports. For a typical preparation, 3.45 g of ZrO_2_ was dispersed in 40 mL of deionized water and stirred until homogenous. Next, 20 mL of a 0.3 M RuCl_3_ aqueous solution were added and stirred for 30 min. Then, 20 mL of 1.5 M aqueous solution of NaBH_4_ was added dropwise to the slurry under vigorous stirring. The molar ratio for NaBH_4_ to Ru(III) was 5/1, which ensured the complete reduction of Ru. The nominal Ru loading were 15 wt% for the catalysts. The black precipitate was washed thoroughly with deionized water until no chloride ions were detectable (0.1 M AgNO_3_ test). The as-prepared catalysts were denoted as CZA, CZB, CZC, and CZD corresponding to the different heterophase structure of ZrO_2_ support, respectively. Ru supported on commercial ZrO_2_ (Ru/C-ZrO_2_) is prepared with the similar method of CZ(A–D) except that the commercial ZrO_2_ is used.

Ru catalysts were prepared with a similar precipitation method described in the literature^35^. 19.46 g RuCl_3_·3H_2_O was dissolved in 200 mL of H_2_O with agitation. 11.12 g NaOH was dissolved in 200 mL of H_2_O and then added to the above stirred solution instantaneously and the resulting mixture was agitated for an additional 30 min. The black precipitate was then transferred into a 1 L Hastelloy autoclave. Hydrogen was introduced into the autoclave to raise the total internal pressure of 5.0 MPa and operated at 150 °C, 800 *rpm* for 3 h. When the reaction mixture was cooled, the resulting black powder was washed with deionized water until Cl^−^ was undetectable, and then the desired Ru catalysts were obtained.

### Characterization

Powder X-ray diffraction (XRD) patterns were performed on a Rigaku Dmax-3C X-ray diffractometer using Cu K_α_ radiation (λ = 0.15418 nm) with a tube voltage of 40 kV and a current of 40 mA. The 2θ angles were scanned from 20 to 80° at 4° min^−1^. Their crystallite sizes were calculated from the peak broadening of the most intense peak (−111) for *m*-ZrO_2_ and (111) *t*-ZrO_2_ according to the Scherrer formula, D = 0.9*λ*/*β* cos θ. Here, *λ* is the X-ray wavelength, and *β* is full width at half maximum. The phase mole ratio of *m*-ZrO_2_ (*X*_m_) and *t*-ZrO_2_ (*X*_t_) in the samples were estimated using the equations proposed by Toraya^37^:


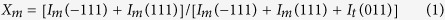



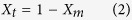


where the *I*_m_(111) and *I*_m_(−111) are the line intensities of the (111) and (−111) peaks for *m*-ZrO_2_, and *I*_t_(011) is the intensity of the (011) peak for *t*-ZrO_2_.

*In situ* Fourier Transform Infrared Spectra (FT-IR) was recorded on Thermo Fisher Nicolet 380 spectrometer with 4 cm^−1^ resolution by signal-averaging over 32 scans. The 100 mg ZrO_2_ sample was finely ground, tableted, and then transferred into an *in situ* vacuum quartz chamber (10 Pa) and dried at 400 °C for 2 h. After cooling to room temperature, the FT-IR spectra were recorded.

The surface morphology and particle size were observed by transmission electron microscopy (TEM) on a JEOL JEM-2011 instrument using an accelerating voltage of 200 kV. The catalyst was dispersed in anhydrous ethanol, sonicated for 2 min, and dripped onto a carbon-film-coated copper grid. A particle size distribution (PSD) histogram was constructed by randomly measuring at least 100 NPs. The multipoint Brunauer−Emmett−Teller surface area (*S*_BET_) and porosity were measured by N_2_ physisorption at 77 K on a Quantachrome NOVA 1000e instrument. Particle size distributions were tested by a laser particle size analyzer (type Rise-2006), which is the most probable distribution. The mean particle size *d*_p_ (m) was observed from the differential curves. Thermal stability of the ZrO_2_ in air was characterized by thermogravimetry-differential scanning calorimetry (TG-DSC) on a NETZSCH STA 449F3 instrument. The surface electronic states were determined by X-ray photoelectron spectroscopy (XPS) on a PHI Quantera SXM spectrometer with Al Kα = 1486.6 eV as the excitation source where the binding energies were calibrated by referencing the C 1 s peak (284.8 eV) to reduce the sample charge effect.

The H_2_ chemisorption was used to determine the dispersion of Ru, which was performed on the Quantachrome Autosorb-IQ gas adsorption analyzer. The weighed sample (~100 mg) was purged with He for 30 min at room temperature and reduced at 200 °C for 2 h under 10% H_2_/Ar. It was then vacuumed for 2 h and cooled to 40 °C. The amount of H_2_ chemisorption was measured under 80, 160, 240, 320, 400, 480, 560, 640, and 720 mm Hg, respectively. The dispersion of Ru was calculated according to the H_2_ uptake with the assumption of H_2_:Ru stoichiometry of 1:2 and a Ru surface with an atomic density of 1.63 × 10^19^ atoms m^−2^ 12.

### Theoretical calculation

The first-principles calculations based on the density functional theory (DFT) offer insight into the different hydrophilicity on ZrO_2_ HSNCs. The *m*-ZrO_2_ and *t*-ZrO_2_ are mainly composed of (−111) and (101) lattice plane, respectively. We only consider and compare the properties of H_2_O molecule adsorbed on these two surfaces. The adsorption energy (*E*_ad_) and the work function (*E*_f_) are calculated with the following expressions:


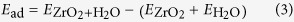



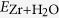
 is the energy of the adsorbed system; 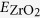 and 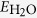 are the energies of the clean surfaces and the single H_2_O molecule, respectively.


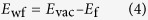


*E*_vac_ and *E*_f_ represent the vacuum level and the Fermi level, respectively.

### Catalytic evaluation

With the as-prepared catalysts, the benzene-selective hydrogenation was carried out in a 1 L Hastelloy autoclave. The autoclave was charged with 4.0 g of catalyst, 47.2 g of ZnSO_4_·7H_2_O (adsorbed Zn^2+^ ions can assist to stabilize a water layer above the catalyst surface)^30^, 280 mL of deionized water (to provide a soluble environment for the additives and assist in forming water/oil interface), and then sealed and purged with H_2_ three times to expel air. The stirring rate was initially fixed at 800 *rpm* with hydrogen pressure of 4.0 MPa. When the temperature reached 150 °C, the line was charged with benzene (140 mL), and the hydrogen pressure was elevated to 5.0 MPa with a stirring rate of 1400 *rpm*. This is sufficient to eliminate the diffusion effects^31^. All reactions were carried out under a kinetically-controlled regime, which is demonstrated in section of *Mass-Transfer Considerations*. The reaction conditions adopted here are typical for selective hydrogenation of benzene^25^,^26^,^27^,^28^,^29^,^30^,^31^,^32^,^33^,^34^,^35^. The reaction process was monitored by discharging ~0.5 mL of the reaction mixture at periodic 5 min followed by analysis on a gas chromatography with a FID detector. Benzene, cyclohexene and cyclohexane were quantified using calibration curves.

To compare the intrinsic catalytic performance, the activity was expressed as the turnover frequency (TOF) of benzene, and the selectivity was expressed as the *S*_40_. Here, *S*_40_ means the value of CHE selectivity when benzene conversion is 40%. To calculate the TOF, we used the specific activity (*r*_0_), i.e., the moles of benzene converted per second at initial reaction time. The experimental benzene content-reaction time (*t*) curve was fitted with a polynomial equation. This was differentiated, and the *r*_0_ was acquired by substituting zero for *t*. Similarly, *r*_obs_ (5, 10, 15, 20, 25) were acquired. The TOF value was calculated using the following equation^12^:


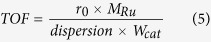


Here, *M*_Ru_ and *W*_cat_ are the molar mass of Ru and the loading of Ru on the catalyst, respectively. The dispersion of Ru was determined by H_2_ chemisorption as described in the Supporting Information.

The methods of Carberry, Wheeler, and Weisz^38^ were used to evaluate the extent of mass-transfer limitations related to diffusion from the liquid to the solid phase and within the catalyst pores. The Carberry number, *Ca*, and the Wheeler-Weisz group, *ηφ*^2^, were calculated with the following expressions:


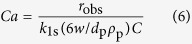



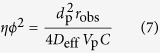


*r*_obs_: observed rate, mol s^−1^; *k*_ls_: liquid/solid mass-transfer coefficient, m s^−1^; *w:* catalyst weight, g; *d*_p_: mean particle size, m; *ρ*_p_: catalyst apparent density, g cm^−3^; *C*: solubility in water of H_2_, C_6_H_6_, C_6_H_10_, mol cm^−3^; *D*_eff_: diffusion coefficient, m^2^ s^−1^; *V*_p_: catalyst volume, cm^3^. The physicochemical data used in calculations are listed in Table S5.

## Results

### Bulk structure, morphology, texture, and the surface properties of the ZrO_2_ HSNCs

The phase mole ratio of *m*-ZrO_2_ versus *t*-ZrO_2_ is related to the concentration of ZrOCl_2_·8H_2_O accompanied by calcination condition (Fig. S1). With the concentration increased from 0.15 to 0.50 mol L^−1^, the phase mole ratios of *m*-ZrO_2_ increased from 60% to 80% (Table S1). Table S1 and Figs S2,3 indicate that the ZrO_2_ HSNCs samples prepared by different concentrations of ZrOCl_2_·8H_2_O have similar crystallite size, particle size distribution, and texture properties. Figure S4 and Table S2 show the effect of calcination time on the texture properties of ZrO_2_. The surface area basically remained unchanged within 2 h calcination. With prolonging calcination time to 3 h, the surface area of the sample decreased to 23 m^2^ g^−1^. The unchanged surface area in the initial calcination time of 2 h is because of an induction period in the phase transformation process.

**Figure 1:**
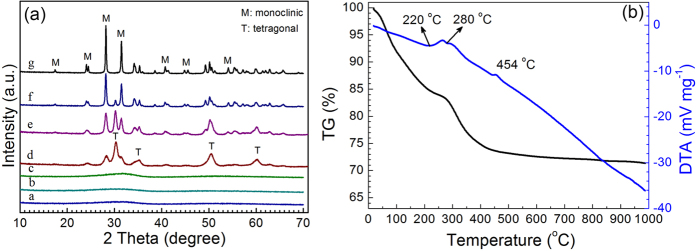
(**a**) XRD patterns of the (*a*) ZrO_2_ precursor, (*b*) powder after azeotropic distillation, and ZrO_2_ HSNCs calcined at (c) 300 °C, (d) 400 °C, (e) 600 °C, (f) 800 °C, and (g) 1000 °C. Synthesis conditions: *n* (NH_4_HCO_3_)/*n* (zirconium ions) = 2; *C*_Zr_ = 0.15 mol L^−1^; pH = 5.1~5.8; *t*_calcination_ = 2 h. (**b**) TG/DTA curves of ZrO_2_ precursor up to 1000 °C in air.

The concentration of ZrOCl_2_·8H_2_O is fixed at 0.15 mol L^−1^ and the calcination time is fixed to 2 h. The ZrO_2_ samples calcined at different temperatures are shown in Fig. 1a. The most striking distinctions between *m*-ZrO_2_ and *t*-ZrO_2_ are the intense peaks at 2*θ* of 28.2° and 31.5° (*m*-ZrO_2_) and the peak at 2*θ* of 30.3° (*t*-ZrO_2_). The phase transformation from *am*-ZrO_2_ to *t*-ZrO_2_ and then to *m*-ZrO_2_ is clearly observed with increasing calcination temperatures. At 400 °C, the diffraction peaks of both *t*-ZrO_2_ and *m*-ZrO_2_ began to appear, and the characteristic peak at 2*θ* of 30.3° (*t*-ZrO_2_) was very high versus peaks at 2*θ* of 28.2° and 31.5° (*m*-ZrO_2_). The *m*-ZrO_2_ and *t*-ZrO_2_ coexisted and the phase proportion of *m*-ZrO_2_ versus *t*-ZrO_2_ increases from ~40% for ZrO_2_(A) to ~90% for ZrO_2_(C) (Table 1). At 1000 °C, the characteristic peak of *t*-ZrO_2_ disappeared entirely, indicating that the *t*-ZrO_2_ converted to *m*-ZrO_2_ completely. This is in accordance with the literature^39^. As the calcination temperature increased, the BET surface area, pore diameter, and pore volume of ZrO_2_ decreased, while the crystallite size increased (Table 1). This is attributable to the conglomerates of crystallites.

Figure 1b shows the TG/DTA curves of the ZrO_2_ precursor up to 1000 °C. The two endothermic peaks in DTA curve at 220 °C and 280 °C indicate the exclusion of structural water of ZrO_2_. The exothermic peak at 454 °C corresponds to the transformation of *t*-ZrO_2_ to *m*-ZrO_2_^40^. The weight loss after 400 °C is minor and is attributed to the dehydroxylation at higher temperatures in air.

**Figure 2:**
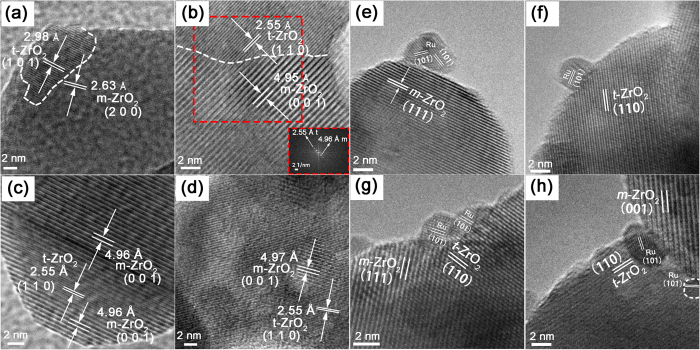
(**a**–**d**) HRTEM images of heterophase-structured ZrO_2_ including the heterophase junction between *m*-ZrO_2_ and *t*-ZrO_2_ obtained from ZrO_2_ (B–D). The inset is FFT images of the red rectangle frame in Fig. 2(b). HRTEM images of the Ru NPs on (**e**) *m*-ZrO_2_ of ZrO_2_(D), (**f**) *t*-ZrO_2_ of ZrO_2_(A), and (**g**,**h**) *m*-ZrO_2_/*t*-ZrO_2_ junction of ZrO_2_(B).

Figure 2a–d gives heterophase structure details of the ZrO_2_ HSNCs. Substantial different lattice fringes were observed, and different interplanar spacing was measured in the ZrO_2_ HSNCs. Two lattice fringes with different interplanar spacing of 2.63 Å (*m*-ZrO_2_) and 2.98 Å (*t*-ZrO_2_) (Fig. 2a), 4.95 Å (*m*-ZrO_2_) and 2.55 Å (*t*-ZrO_2_) (Fig. 2b), 4.96 Å (*m*-ZrO_2_) and 2.55 Å (*t*-ZrO_2_) (Fig. 2c), 4.97 Å (*m*-ZrO_2_) and 2.55 Å (*t*-ZrO_2_) (Fig. 2d) were illustrated—these were ascribed to the (200), (001) planes of *m*-ZrO_2_, and the (101), (110) planes of *t*-ZrO_2_, respectively. Actually, heterophase structures are widely existed in ZrO_2_ HSNCs, and a more representative heterophase structures are revealed in Fig. S5.

When the surface of ZrO_2_ oxidic system is created by truncation of ideal regular extended crystals, chemical bonds are cleaved, and coordinatively unsaturated (*cus*) anions and cations remain exposed in the uppermost layer. If the truncated ZrO_2_ crystallites are exposed to the atmosphere, then both undissociated H_2_O molecules and OH species (dissociated H_2_O) may contribute to the saturation of the *cus* cationic and anionic terminations produced in the outer layer of the ZrO_2_.

**Figure 3:**
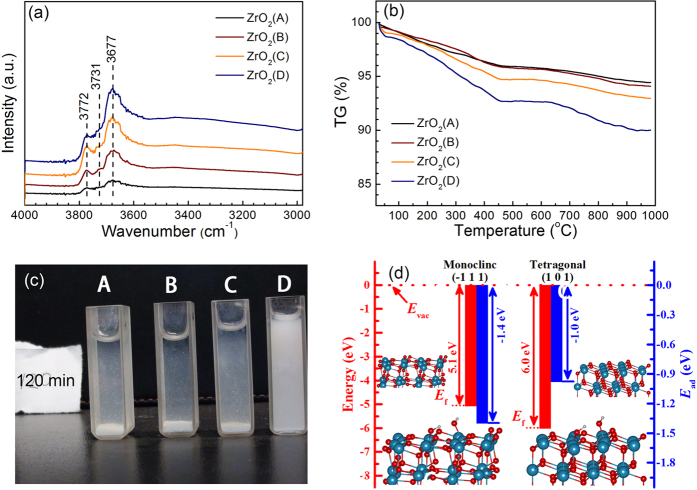
(**a**) *In situ* FT-IR spectra, and (**b**) TG curves of different ZrO_2_ HSNCs in air. (**c**) The sedimentation pictures of ZrO_2_ HSNCs in deionized water at 120 min, A to D means ZrO_2_(A) to ZrO_2_(B). (**d**) *E*_wf_ (red bars) of *m*-ZrO_2_ (−111) and *t*-ZrO_2_ (101), and *E*_ad_ (blue bars) of H_2_O adsorbed on the surfaces. The adsorption structures and clean surfaces are shown inset, the cyan, red, and white atoms denote Zr, O, and H atoms, respectively.

Figure 3a shows the *in situ* FT-IR spectra of ZrO_2_ HSNCs. The samples have been adsorbed and reacted with water in air to reach saturation before test. The bands at 3772 cm^−1^, 3731 cm^−1^ and 3677 cm^−1^ correspond to terminal or monobridged, bi-bridged, and tri-bridged hydroxyl groups of ZrO_2_ HSNCs, respectively^41^. With increasing calcination temperature, the ZrO_2_ transformed from tetragonal to monoclinic phase, and the concentration of ZrO_2_ surface hydroxyl groups increased as shown in Fig. 3a. This is in consistent with the results of literature (the specific case^41^ is seen in EI)^39^,^41^,^42^.

Figure 3b shows the TG curves of the dried ZrO_2_ HSNCs samples. The mass loss increased from 5.6% for ZrO_2_(A) to 9.9% for ZrO_2_(D), which is attributed to the elimination of undissociated H_2_O molecules and OH species on the surface. This further confirms that the *m*-ZrO_2_ possesses more surface hydroxyl groups in the atmosphere. The ZrO_2_ HSNCs sedimentation is tested in water system (Fig. 3c, S6). The settlement from ZrO_2_(A) to ZrO_2_(D) becomes slower. The density and the crystallite size of ZrO_2_ samples are increased from ZrO_2_(A) to ZrO_2_(D), hence the increasing difficulty of sedimentation from ZrO_2_(A) to ZrO_2_(D) were caused by their surface hydrophilicity. The surface hydrophilicity is positively related to the amount of surface hydroxyl groups in water system and reaction condition.

To better understand the different hydrophilicity on ZrO_2_ HSNCs, a first-principles calculations based on the density functional theory (DFT) was performed (Fig. 3d). The *E*_ad_ of *m*-ZrO_2_ (−111) is 0.4 eV larger than that of *t*-ZrO_2_ (101); meanwhile, the relatively smaller *E*_wf_ with higher fermi level of *m*-ZrO_2_ (−111) indicates stronger activity than *t*-ZrO_2_ (101). Therefore, with a more monoclinic phase involved in ZrO_2_ HSNCs, more coordinated water molecules and surface OH groups existed on ZrO_2_ HSNCs.

Hence, the phase mole ratio could be tuned by adjusting the concentration of ZrOCl_2_·8H_2_O as well as the subsequent calcination temperature. The phase mole ratio of *m*-ZrO_2_ versus *t*-ZrO_2_ determined the concentration of ZrO_2_ surface hydroxyl groups and hydrophilicity of the ZrO_2_ samples. This is important for the catalytic performance in benzene-selective hydrogenation that will be elaborated below.

### Ru NPs size distribution, location, and chemical state on ZrO_2_ HSNCs

**Figure 4:**
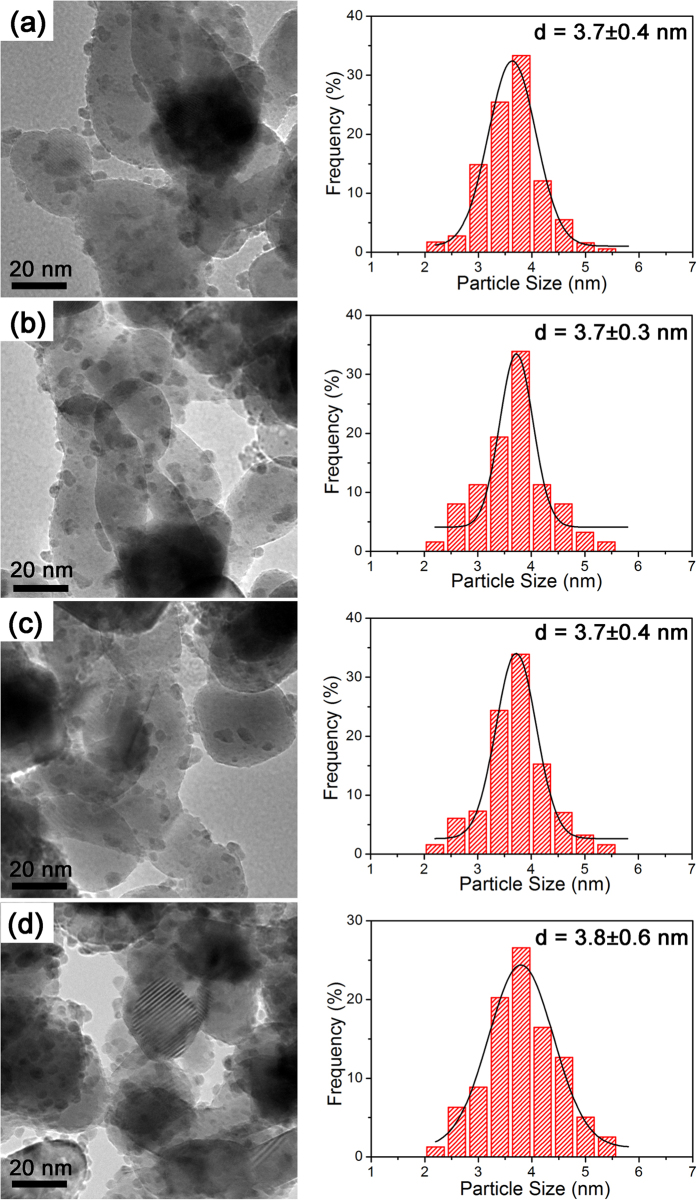
TEM images and PSD histograms with Gaussian analysis fittings of Ru NPs on the (**a**) CZA, (**b**) CZB, (**c**) CZC, and (**d**) CZD. CZA, CZB, CZC, and CZD refer to Ru NPs supported on ZrO_2_(A), ZrO_2_(B), ZrO_2_(C), and ZrO_2_(D), respectively.

Figure 4 shows the TEM images and PSD histograms with Gaussian analysis fittings of the Ru/ZrO_2_ catalysts. The dark Ru NPs displayed narrow PSDs in the range of 2–6 nm with mean particle size centered on 3.7 nm. The Ru NPs located on these ZrO_2_ samples exhibit similar particle size, distribution, and good dispersion behavior. The HRTEM images in Fig. 2e,f show more structural details of these catalysts. The lattice fringe with interplanar spacing values of 2.03 Å is ascribed to the (101) planes of *hcp* Ru, which can be seen in all catalysts. The lattice fringes with interplanar spacing of ~2.85 Å are ascribed to *m*-ZrO_2_ (Fig. 2e); The lattice fringes with interplanar spacing of ~2.55 Å are ascribed to *t*-ZrO_2_ (Fig. 2f), Ru NPs are randomly distributed on the Ru/*m*-ZrO_2_ and Ru/*t*-ZrO_2_ catalysts, respectively (Fig. 2e,f). Figure 4b,c and Fig. S7a–f indicate that the Ru NPs tend to locate at the boundaries of ZrO_2_ HSNCs. The HRTEM images of the CZB catalyst (Fig. 2g,h and Fig. S7g–l) further reveal that the Ru NPs are situated at the *m*-ZrO_2_/*t*-ZrO_2_ junction. The mechanism for the deposition of Ru NPs at the junction will be elaborated below.

These catalysts were further characterized by XPS to probe their electronic characteristics. The stronger Ru 3d peak was not employed to determine the chemical state of Ru for its partial overlapping with C 1 s peak of contaminant carbon. Figure S8 shows the Ru 3p spectra of the catalysts. The Ru 3p_3/2_ BE of 460.4 eV and the 3p_3/2_–3p_1/2_ doublet separation of 22.2 eV evidenced the metallic of Ru in these catalysts^17^. The spectra indicate that Ru NPs supported on ZrO_2_ HSNCs have the same chemical state in the four catalysts.

### Mechanism for the deposition of Ru NPs at the *m*-ZrO_2_/*t*-ZrO_2_ junction

**Figure 5:**
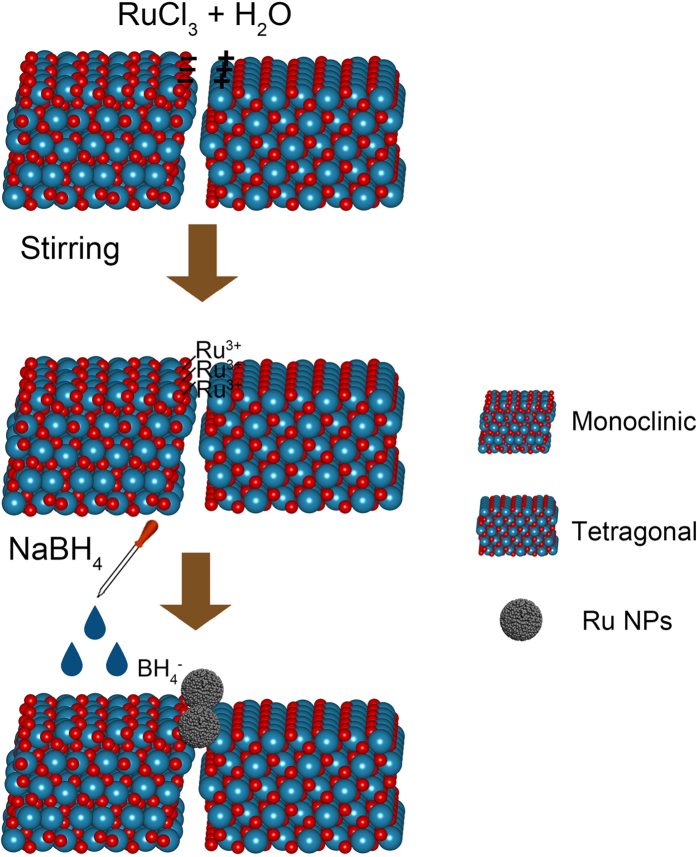
Illustration of the formation mechanism of the Ru NPs situated at the *m*-ZrO_2_/*t*-ZrO_2_ junction via chemical reduction method.

Bell *et al*.^39^,^42^ found that the concentration of the hydroxyl groups and the Zr^4+^/O^2−^ pairs on *m*-ZrO_2_ are both higher than that on *t*-ZrO_2_. Meanwhile, the concentration of O^2−^ anions on *t*-ZrO_2_ is higher than that on *m*-ZrO_2_. When brought into contact, the O^2−^ anions can form hydrogen bonds with the H atoms on *m*-ZrO_2_ at the junction. This leads to *m*-ZrO_2_ with a positive charge and *t*-ZrO_2_ with a negative charge at the junction. Therefore, the Ru^3+^ is preferentially adsorbed on the side of negative charged *m*-ZrO_2_ at the junction. The HRTEM images of the Ru/ZrO_2_ catalyst indicate that the Ru NPs are preferentially situated at the *t*-ZrO_2_/*m*-ZrO_2_ junction. In light of these facts and the mechanism of chemical reduction^43^, the underlying reasons for deposition of Ru NPs at the junction is postulated in Fig. 5. During the chemical reduction process, the negative charged *m*-ZrO_2_ at the junction adsorbed Ru^3+^ and served as the nucleation location; the Ru^3+^ cations are reduced to the Ru0 atoms when the borohydride solution is added. The Ru0 atoms catalyze the decomposition of borohydride to release highly reducing H atoms^43^. The remaining Ru^3+^ cations are more inclined to be reduced at the *t*-ZrO_2_/*m*-ZrO_2_ junction.

## Discussion

### Benzene-selective hydrogenation and implications of the ZrO_2_ HSNCs for the catalytic performance

**Figure 6:**
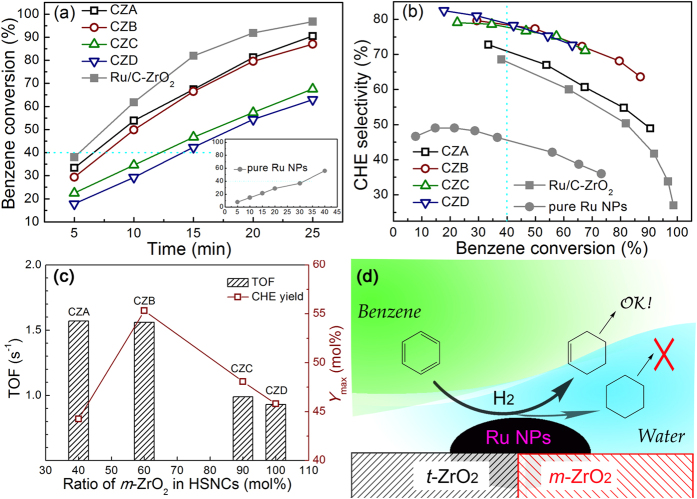
The plots of (**a**) benzene conversion versus time, and (**b**) cyclohexene selectivity versus benzene conversion with CZA, CZB, CZC, CZD, Ru/C-ZrO_2_, and pure Ru NPs catalysts. (**c**) TOF_40_ and *Y*_max_ of catalysts with different phase mole ratios of *m*-ZrO_2_ in HSNCs in benzene-selective hydrogenation to cyclohexene. (**d**) Catalytic mechanism on Ru/ZrO_2_ catalysts.

With the ZrO_2_ samples listed in Table 1 as supports, the Ru/ZrO_2_ catalysts were used for benzene-selective hydrogenation. Figure 6a,b illustrate the hydrogenation of benzene over CZA, CZB, CZC, and CZD. On these catalysts, cyclohexene and cyclohexane are the only products. During the course of the reaction, benzene decreased and cyclohexane increased monotonically. The amount of cyclohexene reached a maximum with the time depending on the type of catalyst. These kinetics obeyed the known behavior of the consecutive reactions.

The catalytic activity and cyclohexene yield of the Ru/ZrO_2_ catalysts are the function of the phase mole ratios (Fig. 6a–c and Table S3). When the ZrO_2_(B) sample with 60% of *m*-ZrO_2_ as supports, the CZB not only exhibits a higher activity (TOF = 1.56 s^−1^), but also shows a high selectivity to cyclohexene (*S*_40_ = 80%). In light of the similarities of the composition, particle size, chemical state, and dispersion of the Ru/ZrO_2_ catalysts verified above. The Ru NPs should not be responsible for the differences in catalytic performances.

Ru particles dispersed in a water/benzene emulsion are predominantly wetted by benzene^44^. However, when the Ru particles are attached to the strongly hydrophilic supports like silica, alumina, or zirconia, the Ru particles become hydrophilic and surrounded by water layer^44^,^45^,^46^. Hronec *et al*.^47^ investigated the benzene-selective hydrogenation performance for Ru-based catalysts supported on strongly hydrophilic resin and hydrophobic charcoal. The results demonstrate that the hydrophilic strength of support controls the hydrophilic/hydrophobic environment around Ru NPs, which determines the catalytic performance of the hydrogenation reaction. The principle of this process is changing Ru NPs from hydrophobic to hydrophilic and controlling cyclohexene mass transport through an aqueous zinc salt solution^27^,^48^,^49^.

Rather, in this work, the superior behavior of the catalyst for benzene-selective hydrogenation to cyclohexene can be explained by the mechanism illustrated in Fig. 6d, S10. The *t*-ZrO_2_ possesses weaker hydrophilicity due to lack of hydroxyl groups, the water layer around catalysts is so thin that benzene is easily adsorbed on the Ru NPs easily. The formed cyclohexene is prone to re-adsorption and is hydrogenated to cyclohexane on the surface of Ru NPs (Fig. S10a). The cyclohexane becomes the primary product (*S*_40_ = 71%) with a high activity (TOF = 1.57 s^−1^). *m*-ZrO_2_ possesses strong hydrophilicity due to more surface hydroxyl groups. When *m*-ZrO_2_ phase appeared in ZrO_2_ HSNCs, the water layer around Ru NPs becomes heterogeneous (Fig. 6d). This leads to the greatly increased cyclohexene selectivity on CZ(B-C) (*S*_40_ ≈ 80%), and relatively high activity (TOF = 1.56 s^−1^) for CZB. On the pure *m*-ZrO_2_, the water layer around Ru NPs is so thick that benzene is hindered to diffuse through the water layer onto the catalyst surface (Fig. S10c), resulting in a high cyclohexene selectivity (*S*_40_ = 80%) and a very low activity (TOF = 0.93 s^−1^).

The water-solubility of benzene (12 times) and cyclohexene (2 times) are higher than that of cyclohexane^38^. The hydrophilic stagnant water layer causes cyclohexene to diffuse from catalyst surface to the organic phase, and prevent cyclohexene from further hydrogenation. The water layer concept is widely recognized in benzene hydrogenation reaction system^30^. When the support has strong/weak hydrophilic surface, it is reasonable to deduce there existed a specific diffusion-restricted area formed on the *m*-ZrO_2_/*t*-ZrO_2_ junction of the catalysts. The video (in the SI) shows that the Ru catalysts are surrounded by a water layer in a simulated reaction condition, demonstrating that water layers are around the Ru-based catalysts during reaction.

On the other hand, if Ru NPs are not armed with ZrO_2_ HSNCs, the heterogeneous strong/weak hydrophilic interface cannot be formed. There was a large amount of benzene adsorption, activation, and hydrogenation on Ru NPs without the desired cyclohexene selectivity and yield due to the nature of surface hydrophobicity of Ru NPs (Fig. 6a,b, Fig. S9, and Table S3). Furthermore, even if the Ru NPs supported on commercial ZrO_2_ without heterophase structures, it is still hard to get a satisfied cyclohexene selectivity and yield (Fig. 6a,b and Table S3). The texture properties of commercial ZrO_2_ is seen in Fig. S11 and Table S4.

### Mass-Transfer Considerations

Catalytic reactions in an autoclave reactor involve processes such as gas to liquid, liquid to solid particle mass transfer, intraparticle diffusion, adsorption, surface reaction, and desorption of products. To evaluate the extent of mass-transfer limitations related to diffusion from the liquid to the surface of solid phase and within the catalyst particles, the methods introduced by Carberry, Wheeler and Weisz have been adopted^38^.

The Carberry number, *Ca*, repreAs shown in Table 2 and Table S6, the Carberry number and Wheeler-Weisz group are very small (*Ca* < 0.05, *ηφ*^2^ < 0.1) at all reaction times for the four catalysts. This indicates that liquid-solid mass transfer and pore diffusion resistance of the reaction rate can be neglected^38^. Therefore, the reactions were carried out under a kinetically-controlled regime^43^,^44^.

### Comparison with CZB with other reported supported catalysts

**Figure 7:**
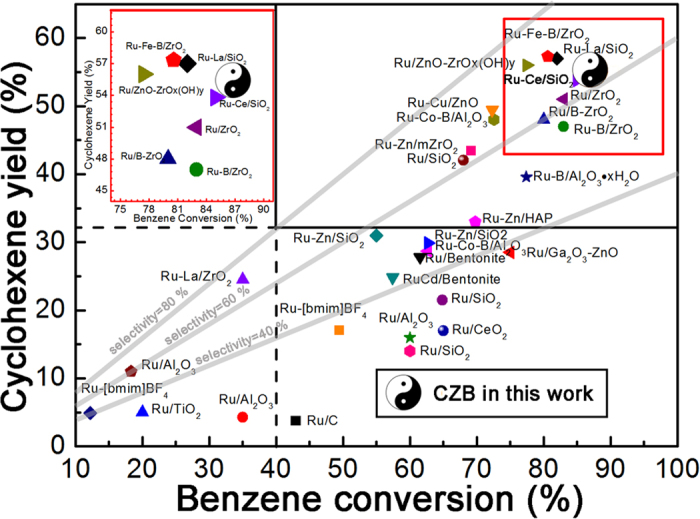
The catalytic performance of benzene-selective hydrogenation over CZB compared with other literature results; the inset is the larger version of results in the red box. Detailed information (values of conversion, selectivity, yield and references) is listed in Table S7.

To date, it is difficult to improve cyclohexene selectivity at a relatively high activity effectively. Achieving a high yield of cyclohexene from the benzene-selective hydrogenation remains a challenge. In Fig. 7 the most important supported Ru-based catalysts results are summarized in terms of cyclohexene yield versus benzene conversion. All data were obtained at the typical condition for the partial hydrogenation with similar catalyst loading, 140–150 °C, and 3–5 MPa H_2_ pressure. The diffusion effect was excluded through high stirring speed.

In principle, only when benzene conversion is larger than 40% with cyclohexene selectivity higher than 80% at the same time (i.e. the yield >32%), can the catalyst be industrialized for benzene-selective hydrogenation (solid black line box). The as-prepared CZB undoubtedly achieves a high conversion of 87% and product yield of 55.3%. This is quite comparable to Ru-supported catalysts including Al_2_O_3_, SiO_2_, C, TiO_2_, CeO_2_, ZrO_2_, ZnO, Ga_2_O_3_, HAP, and bentonite as supports.

## Conclusions

The ZrO_2_ HSNCs were synthesized with tunable ratios of *m*-ZrO_2_ versus *t*-ZrO_2_ by adjusting the synthesis parameters and calcination conditions. They were used as effective supports to fabricate heterophase Ru/ZrO_2_ catalysts for benzene-selective hydrogenation. The as-prepared CZB achieves a high conversion of 87.0% and cyclohexene yield of 55.3%. This is quite comparable to other reported Ru-supported catalysts. The ZrO_2_ HSNCs possesses more appropriate surface hydroxyl groups and surface properties than their single-phase counterparts. The excellent catalytic performances with high activity and selectivity are attributed to the specific diffusion-restricted area formed at the *m*-ZrO_2_/*t*-ZrO_2_ junction. The synthesis strategy and tuning approach will be useful for the design of supported Ru-based catalysts for benzene-selective hydrogenation to cyclohexene and other difficult catalytic reactions. This work provides an outstanding example of ZrO_2_ HSNCs as a support for fabrication of heterogeneous catalysts.

## Additional Information

**How to cite this article:** Peng, Z. *et al*. Heterophase-structured nanocrystals as superior supports for Ru-based catalysts in selective hydrogenation of benzene. *Sci. Rep.* **7**, 39847; doi: 10.1038/srep39847 (2017).

**Publisher's note:** Springer Nature remains neutral with regard to jurisdictional claims in published maps and institutional affiliations.

## Supplementary Material

Supplementary Information

Supplementary Video S1

## Figures and Tables

**Table 1:** The phase mole ratios of *m*-ZrO_2_ versus *t*-ZrO_2_ and the textural properties of the ZrO_2_ HSNCs^a^.

Sample^b^	Ratios of *m*- versus *t*-ZrO_2_^c^	Crystallite size^d^ (nm)	*S*_BET_ (m^2^·g^−1^)	*D*_pore_ (nm)	*V*_Total_ (cm^3^·g^−1^)
ZrO_2_(A)	4:6	17	51	15.1	0.38
ZrO_2_(B)	6:4	22	39	11.7	0.23
ZrO_2_(C)	9:1	35	35	10.8	0.06
ZrO_2_(D)	10:0	43	18	7.9	0.03

^a^Synthesis conditions: *n* (NH_4_HCO_3_)/*n* (zirconium ions) = 2; pH = 5.1~5.8; *C*_Zr_ =  0.15 mol L^−1^; pH = 5.1~5.8; *t* = 2 h.

^b^ZrO_2_(A), ZrO_2_(B), ZrO_2_(C), and ZrO_2_(D) are calcined at 400, 600, 800, and 1000 °C, respectively.

^c^According to the equations proposed by Toraya^37^.

^d^According to the Scherrer equation.

**Table 2:** Rate of benzene disappearance, Carberry number (*Ca*) and Wheeler-Weisz group (*ηφ* ^2^) over CZB at all reaction times.

Time (min)	*r*_obs_ (C_6_H_6_)×10^3^ (mol s^−1^)	*Ca* (C_6_H_6_) ×10^3^	*C*a (C_6_H_10_) ×10^2^	*Ca* (H_2_) ×10^3^	*ηφ*^2^ (C_6_H_6_) ×10^2^	*ηφ*^2^ (C_6_H_10_) ×10	*ηφ*^2^ (H_2_) ×10^2^
5	1.30	0.45	0.28	0.79	0.19	0.12	0.27
10	1.04	0.36	0.23	0.64	0.15	0.09	0.21
15	0.77	0.27	0.17	0.47	0.11	0.07	0.16
20	0.51	0.18	0.11	0.31	0.07	0.05	0.10
25	0.25	0.09	0.05	0.15	0.04	0.02	0.05

The Carberry number, *Ca*, represents the extent of external mass-transfer limitation, and Carberry numbers smaller than 0.05 indicates that the diffusion retardation by external mass transfer may be neglected. The Wheeler and Weisz group, *ηφ*^2^, represents the extent of pore diffusion limitation and values smaller than 0.1 means that the pore diffusion limitation is negligible.
