# Supplementary material for: Heterophase-structured nanocrystals as superior supports for Ru-based catalysts in selective hydrogenation of benzene
Source: Sci Rep. 2017 Jan 6;7:39847. doi: 10.1038/srep39847 (PMC5216362; doi:10.1038/srep39847)
Supplement: Supplementary Information [file srep39847-s1.doc]

**SUPPORTING INFORMATION (SI)**

**Heterophase-structured nanocrystals as superior supports for Ru-based catalysts in selective hydrogenation of benzene**

Zhikun Peng, Xu Liu, Shuaihui Li, Zhongjun Li, Baojun Li,* Zhongyi Liu* and Shouchang Liu

School of Chemistry and Molecular Engineering, Zhengzhou University, 100 Kexue Avenue, Zhengzhou 450001, China

* Corresponding author. Tel: +86-371-67783384, Email: liuzhongyi@zzu.edu.cn (Zhongyi Liu); lbjfcl@zzu.edu.cn (Baojun Li).

**Figure S1.** XRD patterns of ZrO_2_ prepared with different concentrations of ZrOCl_2_·8H_2_O. (a) 0.15 mol·L^–1^, (b) 0.3 mol·L^–1^, and (c) 0.5 mol·L^–1^. Synthesis conditions: *n* (NH_4_HCO_3_)/*n* (zirconium ions) = 2; pH = 5.1~5.8; calcination temperature (*T*) = 600 °C; calcination time (*t*) = 2 h.

Fig. S1 shows the powder XRD patterns of the ZrO_2_ prepared with 0.15, 0.3 and 0.5 mol·L^–1^ ZrOCl_2_·8H_2_O, respectively. The most intensive peak at the 2*θ* of 30.3° is characteristics of (011) plane of *t*-ZrO_2_, and the most intensive peaks at the 2*θ* of 28.2° and 31.4° are attributed to (−111) and (111) planes of *m*-ZrO_2_, respectively.

**Table S1**

The phase mole ratios of *m*-ZrO_2_ versus *t*-ZrO_2_, their crystallite sizes and textural parameters of ZrO_2_ prepared with different concentrations of ZrOCl_2_·8H_2_O.*^a^*

| *C*_ZrOCl2_ (mol·L^–1^) | Ratios of *m*-ZrO_2_ versus *t*-ZrO_2_^b^ | Crystallite size*^c^* (nm) | *S*_BET_ (m^2^·g^–1^) | *D*_pore_ (nm) | *V*_Total_ (cm^3^·g^–1^) |
| --- | --- | --- | --- | --- | --- |
| 0.15 | 6:4 | 22 | 39 | 11.7 | 0.23 |
| 0.30 | 7:3 | 26 | 46 | 6.2 | 0.14 |
| 0.50 | 8:2 | 22 | 40 | 9.6 | 0.19 |

*^a^* Synthesis conditions: *n* (NH_4_HCO_3_)/*n* (zirconium ions) = 2; pH = 5.1~5.8; *T =* 600 °C; calcination time (*t*) = 2 h.

*^b^* According to the equations proposed by Toraya.^1^

*^c^* According to the Scherrer equation.

**Figure S2.** The particle size distribution curves of the ZrO_2_ prepared with different ZrOCl_2_·8H_2_O concentrations. Synthersis conditions: *n* (NH_4_HCO_3_)/*n* (zirconium ions) = 2; pH = 5.1~5.8; *T =* 600 °C; calcination time (*t*) = 2 h.

**Figure S3.** The (a) N_2_ adsorption-desorption isotherms and (b) pore size distribution curves of ZrO_2_ prepared with different concentrations of ZrOCl_2_·8H_2_O. Synthersis conditions: *n* (NH_4_HCO_3_)/*n* (zirconium ions) = 2; pH = 5.1~5.8; *T =* 600 °C; calcination time (t) = 2 h.

Table S1 indicates that the phase mole ratio of *m*-ZrO_2_ to *t*-ZrO_2_ is a strong function of the concentration of ZrOCl_2_·8H_2_O. The ZrO_2_ consist of polycrystalline porous conglomerates of crystallites with diameters about 20 nm. The ZrO_2_ particles, in turn, have diameters of 2 to 6 μm, as found from particle size distribution analysis (Fig. S2). The adsorption isotherms are attributed to type IV with typical hysteresis loops of type H1 defined by IUPAC. The relative pressures p/p^0^ at the separating site of the adsorption and desorption isotherms are beyond 0.8, indicating that the samples are powders with mesopores and bigger pores (Fig. S3a). The pore size distribution ranges from 10 to 60 nm for the ZrO_2_ prepared with different concentrations of ZrOCl_2_·8H_2_O (Fig. S3b). All of these implies that the concentrations of ZrOCl_2_·8H_2_O mainly affect the phase mole ratio of *m*-ZrO_2_ to *t*-ZrO_2_.

**Figure S4.** The (a) N_2_ adsorption (close symbol)-desorption (open symbol) isotherms and (b) pore size distribution curves of ZrO_2_ calcined for different times at 600 °C. Synthersis conditions: ZrOCl_2_·8H_2_O = 0.15 mol L^–1^, *n* (NH_4_HCO_3_)/*n* (zirconium ions) = 2; pH = 5.1~5.8.

The textural parameters of ZrO_2_ calcinated at 600 °C for different times were also examined (Fig. S4 and Table S2). The type of the adsorption-desorption isotherms and the hysteresis loops as well as the pore size distribution for the ZrO_2_ calcination within 2 h basically remained unchanged (Fig. S4). While the calcination time increased to 3 h, the surface area of the sample decreased to 23 m^2^ g^–1^. Therefore, it is experimentally considered the calcinations time of 2 h is more suitable.

**Table S2.**

The textural parameters of ZrO_2_ calcined for different times at 600 °C.*^a^*

| Calcination time (h) | BET surface area (m^2^ g^–1^) | Average pore diameter (nm) | Total pore volume (cm^3^·g^–1^) |
| --- | --- | --- | --- |
| 1 | 40 | 9.6 | 0.19 |
| 2 | 39 | 11.7 | 0.23 |
| 3 | 23 | 15.7 | 0.18 |

*^a^* Synthersis conditions: ZrOCl_2_·8H_2_O = 0.15 mol L^–1^, *n* (NH_4_HCO_3_)/*n* (zirconium ions) = 2; pH = 5.1~5.8.


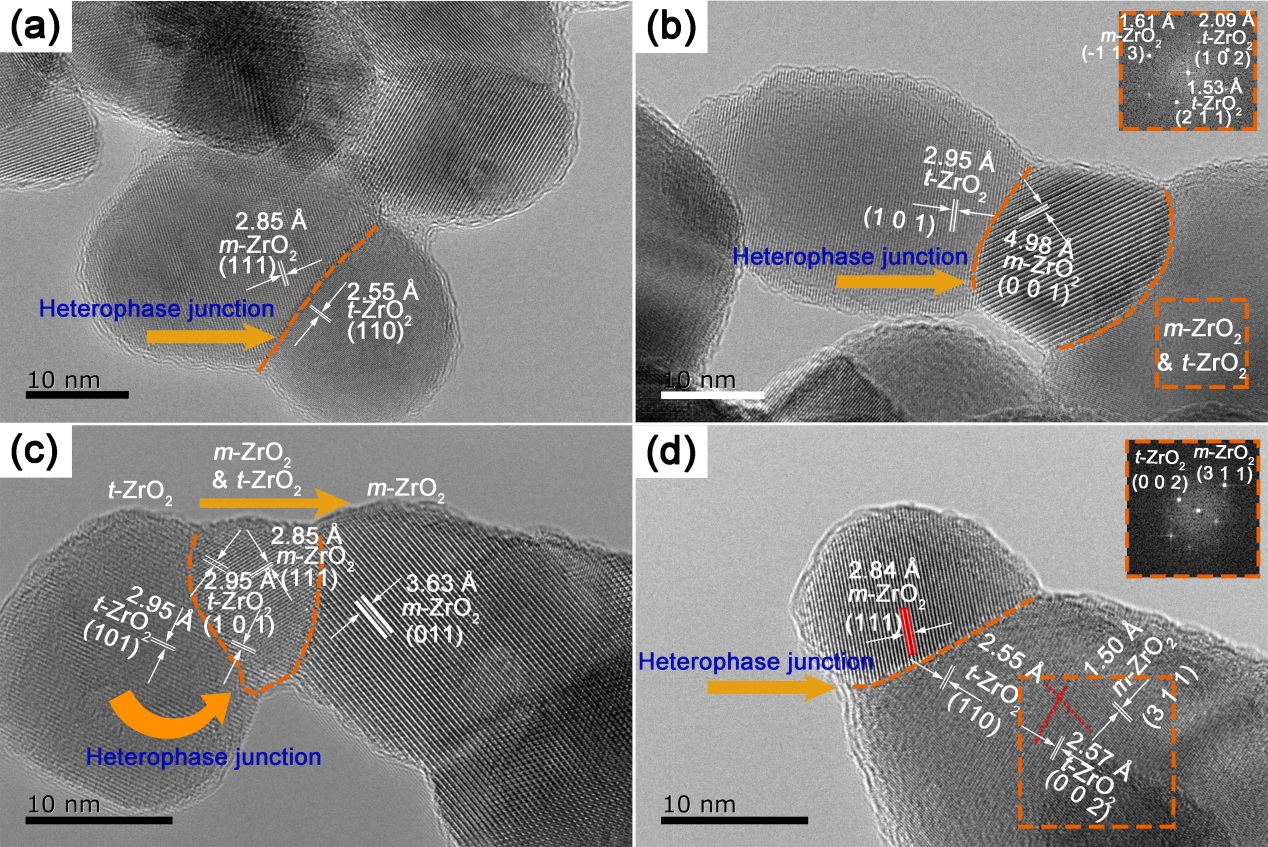


**Figure S5.** HRTEM images heterophase structured of ZrO_2_(B), the inset is FFT images of corresponding rectangle frame in Fig. (b) and (d).

Specifically in literature 41, the amorphous ZrO_2_ powder calcinated at 600 °C (denoted as ZRP873) and 800 °C (denoted as ZRP1073), and they possess a ~70% and ~90% monoclinic phase mole ratio, respectively. The ZRP1073 sample has a higher concentration of hydroxyl groups than ZRP873 after vacuum dehydration for 1 h at 200 °C and 400 °C.


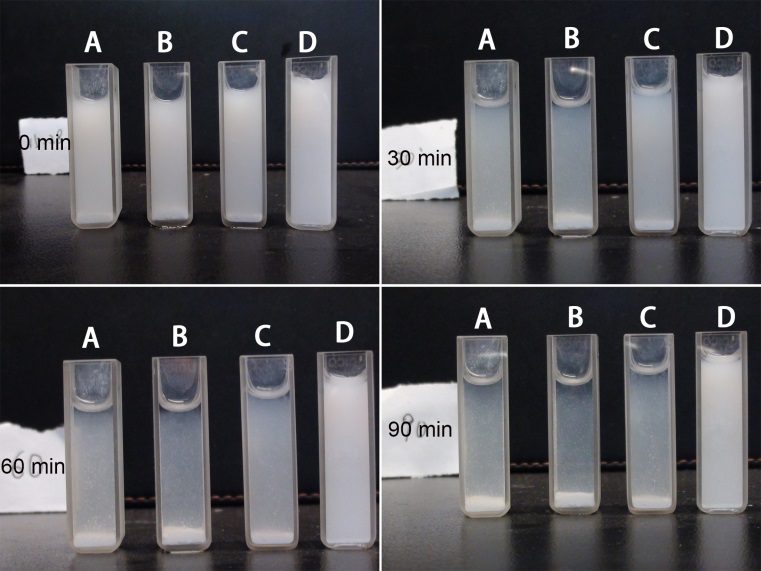


**Figure S6.** The sedimentation pictures of ZrO_2_ in deionized water every 30 min. A, B, C, and D mean ZrO_2_(A), ZrO_2_(B), ZrO_2_(B), and ZrO_2_(D), respectively. The samples are dispersed in water under sonication for 30 min at the beginning.


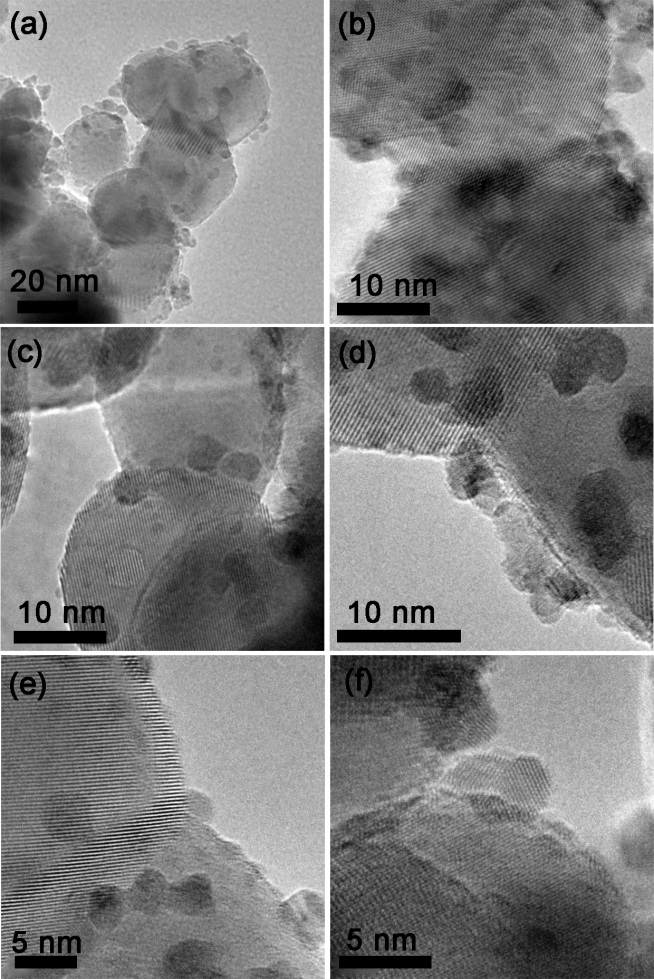


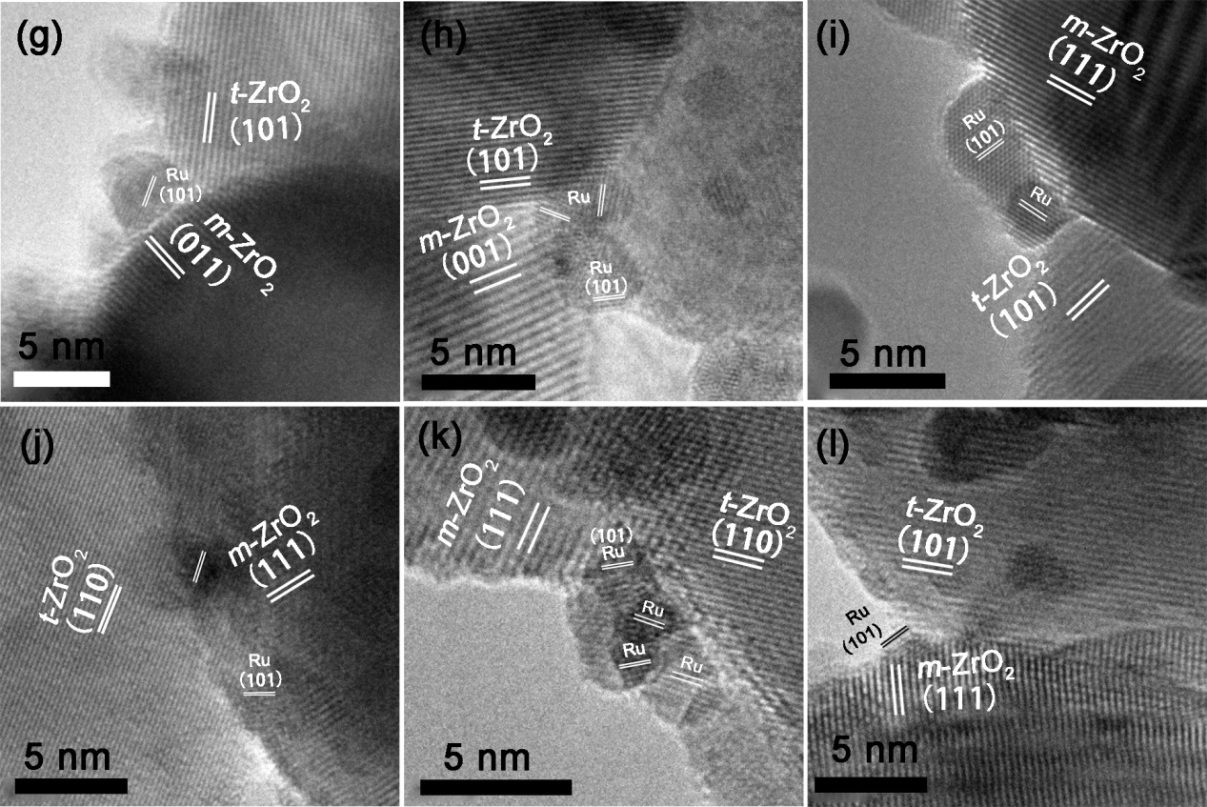


**Figure S7.** (a–f) TEM and (g–l) HRTEM images of the Ru NPs bridge on *m*-ZrO_2_/*t*-ZrO_2_ for ZrO_2_(B).

**Figure S8.** Ru 3p XPS spectra of the (a) Ru/ZrO_2_(A), (b) Ru/ZrO_2_(B), (c) Ru/ZrO_2_(C), (d) Ru/ZrO_2_(D).

**Figure S9.** The plots of (a) benzene conversion versus time, and (b) cyclohexene selectivity versus benzene conversion over CZB, and Ru NPs with different loadings.

| **Table S3.**  Results of the partial hydrogenation of benzene over the Ru/ZrO_2_ catalysts.^a^   | Catalysts | *r*_0_^b^ | TOF (s^–1^) | *S*_40_ (mol%)^c^ | *Y*_max_ (mol%) | Dispersion^d^  (%) | | --- | --- | --- | --- | --- | --- | | CZA | 1.61 | 1.57 | 71 | 44.2 | 19.9 | | CZB | 1.56 | 1.56 | 80 | 55.3 | 19.4 | | CZC | 1.00 | 0.99 | 79 | 48.1 | 19.7 | | CZD | 0.86 | 0.93 | 80 | 45.8 | 18.0 | | pure Ru^e^ | n. m.^j^ | n. m.^j^ | 45 | 26.4 | n. m.^j^ | | Ru/C-ZrO_2_^f^ | n. m.^j^ | n. m.^j^ | 67 | 41.2 | n. m.^j^ | |
| --- | --- | --- | --- | --- | --- | --- | --- | --- | --- | --- | --- | --- | --- | --- | --- | --- | --- | --- | --- | --- | --- | --- | --- | --- | --- | --- | --- | --- | --- | --- | --- | --- | --- | --- | --- | --- | --- | --- | --- | --- | --- | --- |

*^a^* Reaction conditions: 140 mL of benzene, 4.0 g of catalyst, 47.2 g of ZnSO_4_·7H_2_O, 280 mL of H_2_O, temperature 150 °C, 5.0 MPa H_2_ pressure, and stirring rate of 1400 *rpm*.

^b^ Initial specific activity of benzene in mmol s^−1^.

^c^ Selectivity toward cyclohexene when benzene conversion is at 40 mol%.

^d^ Dispersion of the Ru NPs determined by H_2_ chemisorption.

^e^ Using 0.2 g of Ru catalyst.

^f^ Using the commercial ZrO_2_ as a support for comparison.

^j^ Not measured.


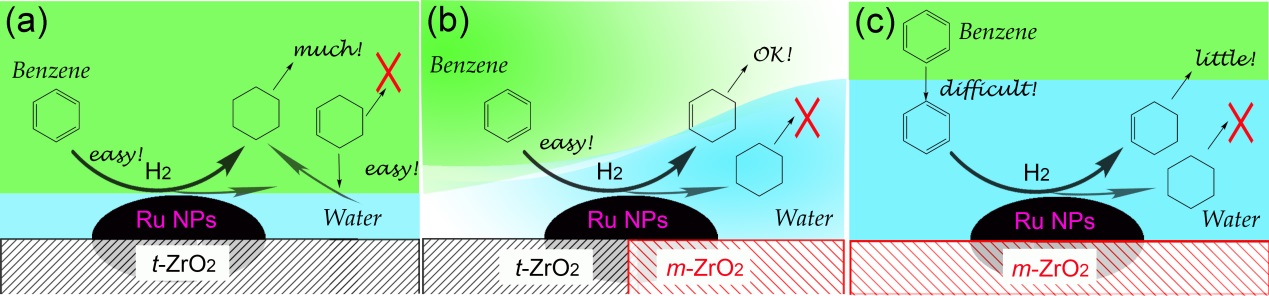


**Figure S10.** (a-c) Schematic illustration of the mechanism for benzene hydrogenation on the different hydrophilicity ZrO_2_ support.

**Video S1.** The water layer around Ru-based catalysts in a simulated reaction condition.

**Figure S11.** (a) N_2_ adsorption-desorption isotherms and (b) the corresponding Barrett-Joyner-Halenda (BJH) pore size distribution curves of commercial ZrO_2_.

**Table S4.** Textural properties of commercial ZrO_2_.

| Sample | *S*_BET_ (m^2^·g^-1^) | *V*_Pore_ (cm^3^/g) | *D*_Pore_ (nm) |
| --- | --- | --- | --- |
| Commercial ZrO_2_ | 34 | 0.13 | 7.8 |

**Table S5.**

Physicochemical data used in the Mass-Transfer calculations.^3^

|  | Values and units | Measuring method |
| --- | --- | --- |
| *k*_1s_ of hydrogen | 3.8×10^-3^ m/s | 3 |
| *k*_1s_ of benzene | 1.8×10^-3^ m/s | 3 |
| *k*_1s_ of cyclohexene | 1.7×10^-3^ m/s | 3 |
| *C* of hydrogen | 3.2×10^-5^ mol/cm^3^ | pure component |
| *C* of benzene | 1.2×10^-4^ mol/cm^3^ |
| *C* of cyclohexene | 2.0×10^-5^ mol/cm^3^ |
| *D*_eff_ of hydrogen | 2.0×10^-8^ m^2^/s | in water |
| *D*_eff_ of benzene | 7.6×10^-9^ m^2^/s |
| *D*_eff_ of cyclohexene | 7.4×10^-9^ m^2^/s |
| *d*_p_ | 2.2×10^-6^ m | (distribution 0.2−8 µm) ^a^ |
| *w* | 4.0 g | b |
| *ρ*_p_ | 0.81 g/cm^3^ | c |
| *V*_p_ | 0.92 cm^3^ | d |

^a^ Measured by a particle size analyzer.

^b^ The active component weight in catalyst (g)

^c^ Calculated from the catalyst weight divided by the catalyst particle volume.

^d^ Calculated from the total pore volume of the support (0.23 cm^3^/g), which was measured by the N_2_ physisorption.

**Table S6**.

Rate of benzene disappearance, Carberry number (*Ca*) and Wheeler-Weisz group (*ηφ*^2^):

over the CZA at all reaction times

| Time (min) | *r*_obs_ (C_6_H_6_)×10^3^ (mol/s) | *Ca* (C_6_H_6_) ×10^3^ | Ca (C_6_H_10_) ×10^2^ | *Ca* (H_2_) ×10^3^ | *ηφ*^2^ (C_6_H_6_) ×10^2^ | *ηφ*^2^ (C_6_H_10_) ×10 | *ηφ*^2^ (H_2_) ×10^2^ |
| --- | --- | --- | --- | --- | --- | --- | --- |
| 5 | 1.28 | 0.44 | 0.28 | 0.78 | 0.18 | 0.11 | 0.26 |
| 10 | 1.06 | 0.36 | 0.23 | 0.65 | 0.15 | 0.09 | 0.22 |
| 15 | 0.85 | 0.29 | 0.18 | 0.52 | 0.12 | 0.08 | 0.17 |
| 20 | 0.64 | 0.22 | 0.14 | 0.39 | 0.09 | 0.06 | 0.13 |
| 25 | 0.42 | 0.14 | 0.09 | 0.26 | 0.06 | 0.04 | 0.09 |

over CZC at all reaction times

| Time  (min) | *r*_obs_ (C_6_H_6_)×10^3^  (mol/s) | *Ca* (C_6_H_6_)  ×10^3^ | Ca (C_6_H_10_)  ×10^2^ | *Ca* (H_2_)  ×10^3^ | *ηφ*^2^ (C_6_H_6_)  ×10^2^ | *ηφ*^2^ (C_6_H_10_)  ×10 | *ηφ*^2^ (H_2_)  ×10^2^ |
| --- | --- | --- | --- | --- | --- | --- | --- |
| 5 | 0.87 | 0.30 | 0.19 | 0.53 | 0.13 | 0.08 | 0.18 |
| 10 | 0.75 | 0.26 | 0.16 | 0.46 | 0.11 | 0.07 | 0.15 |
| 15 | 0.62 | 0.21 | 0.14 | 0.38 | 0.09 | 0.06 | 0.13 |
| 20 | 0.50 | 0.17 | 0.11 | 0.31 | 0.07 | 0.04 | 0.10 |
| 25 | 0.37 | 0.13 | 0.08 | 0.23 | 0.05 | 0.03 | 0.08 |

over CZD at all reaction times

| Time  (min) | *r*_obs_ (C_6_H_6_)×10^3^  (mol/s) | *Ca* (C_6_H_6_)  ×10^3^ | Ca (C_6_H_10_)  ×10^2^ | *Ca* (H_2_)  ×10^3^ | *ηφ*^2^ (C_6_H_6_)  ×10^2^ | *ηφ*^2^ (C_6_H_10_)  ×10 | *ηφ*^2^ (H_2_)  ×10^2^ |
| --- | --- | --- | --- | --- | --- | --- | --- |
| 5 | 0.78 | 0.27 | 0.17 | 0.48 | 0.11 | 0.07 | 0.16 |
| 10 | 0.70 | 0.24 | 0.15 | 0.43 | 0.10 | 0.06 | 0.14 |
| 15 | 0.62 | 0.21 | 0.14 | 0.38 | 0.09 | 0.05 | 0.13 |
| 20 | 0.54 | 0.19 | 0.12 | 0.33 | 0.08 | 0.05 | 0.11 |
| 25 | 0.45 | 0.15 | 0.10 | 0.28 | 0.06 | 0.04 | 0.09 |

**Table S7.**

Detailed information of catalysts shown in Table 1, with respective references.

| Catalyst | Benzene Conv. (%) | Cyclohexene Select. (%) | Cyclohexene Yield (%) | Reference |
| --- | --- | --- | --- | --- |
| Ru/C | 43.0 | 8.6 | 3.7 | 4 |
| Ru/Al_2_O_3_ | 35.0 | 12.3 | 4.3 | 5 |
| RuFe/TiO_2_ | 20.0 | 25.0 | 5.0 | 6 |
| Ru-Cd/Bentonite | 57.4 | 43.1 | 24.8 | 7 |
| Ru-Co-B/Al_2_O_3_ | 62.7 | 45.7 | 28.7 | 8 |
| Ru/ZnO-ZrO_x_(OH)_y_ | 77.5 | 72.3 | 56.0 | 9 |
| Ru-[bmim]BF_4_ | 12.2 | 40.5 | 4.9 | 10 |
| Ru/Al_2_O_3_ | 18.3 | 60.0 | 11.0 | 11 |
| Ru/SiO_2_ | 60.0 | 23.3 | 14.0 | 12 |
| Ru/Al_2_O_3_ | 60.0 | 26.7 | 16.0 | 13 |
| Ru/CeO_2_ | 65.0 | 26.2 | 17.0 | 14 |
| Ru-[bmim]BF_4_ | 49.5 | 34.1 | 17.0 | 15 |
| Ru/SiO_2_ | 64.9 | 33.1 | 21.5 | 16 |
| Ru-La/ZrO_2_ | 35.0 | 70.0 | 24.5 | 17 |
| Ru/Bentonite | 61.5 | 45.2 | 27.8 | 18 |
| Ru/Ga_2_O_3_-ZnO | 75.0 | 38.0 | 28.5 | 19 |
| Ru-Zn/SiO_2_ | 62.7 | 47.7 | 29.9 | 20 |
| Ru-Zn/SiO_2_ | 55.0 | 56.4 | 31.0 | 21 |
| Ru-Zn/HAP | 69.8 | 47.3 | 33.0 | 22 |
| Ru-Co-B/Al_2_O_3_ | 72.6 | 48.0 | 34.8 | 23 |
| Ru-B/Al_2_O_3_·*x*H_2_O | 77.4 | 51.2 | 39.6 | 24 |
| Ru/SiO_2_ | 68.0 | 63.0 | 42.0 | 25 |
| Ru-Zn/*m*-ZrO_2_ | 69.2 | 62.7 | 43.4 | 26 |
| Ru-B/ZrO_2_ | 83.0 | 56.0 | 47.0 | 27 |
| Ru/B-ZrO_2_ | 80.0 | 60.0 | 48.0 | 28 |
| Ru-Cu/ZnO | 72.3 | 68.3 | 49.4 | 29 |
| Ru/ZrO_2_ | 83.0 | 62.0 | 51.0 | 30 |
| Ru-Ce/SiO_2_ | 85.0 | 63.3 | 53.8 | 31 |
| Ru-La/SiO_2_ | 82 | 69 | 57 | 32 |
| Ru-Fe-B/ZrO_2_ | 80.6 | 71.1 | 57.3 | 33 |

References

1 Meskin, P. E., *et al.* *Ultrason. Sonochem.* **13**, 47−53 (2006).

2 Sun, H. J *et al.* *Appl. Catal. A: Gen.* **450**, 160−168 (2013).

3 Hu, S. C. & Chen, Y. W. Ind. *Eng. Chem. Res.* **36**, 5153−5159 (1997).

4 Zanutelo, C., Landers, R., Carvalho, W. A. & Cobo, A. J. G. *Appl. Catal. A:Gen.* **409-410**, 174−180 (2011).

5 Rodrigues, M. F. F. & Cobo, A. J. G. *Catal. Today.* **149**, 321 (2010).

6 Da-Silva, J. W. & Cobo, A. J. G. *Appl. Catal. A: Gen.* **252**, 9 (2003).

7 Wang, W. T. *et al.* *ChemCatChem*, **4**, 1836 (2012).

8 Fan, G. Y. *et al.* *Catal. Commun.* **10**, 98 (2008).

9 Liu, H. Z. *et al.* *Green Chem.* **13**, 1106 (2011).

10 Quin, Y. *et al.* *Chin. J. Catal.* **32**, 1727 (2011).

11 Schwab, F., Lucas, M. & Claus, P. *Angew. Chem. Int. Ed.* **50**, 10453 (2011).

12 Spinacé, E. V. & Vaz, J. M. *Catal. Commun.* **4**, 91 (2003).

13 Suppino, R. S., Landers, R. & Cobo, A. J. G. *Appl Catal A: Gen.* **452**, 9 (2013).

14 Zonetti, P. D. C., Landers, R. & Cobo, A. J. G. *Appl. Surf. Sci.* **254**, 6849 (2008).

15 Xue, W. *et al.* *Chin. J. Catal.* **33**, 1913 (2012).

16 Bu, J. *et al.* *Stud. Surf. Sci. Catal.* **165**, 769 (2007).

17 Liu, S. *et al.* *Nat. Gas. Chem.* **14**, 226 (2005).

18 Wang, W. T. *et al.* *J Mol Catal A: Chem.* **355**, 174 (2012).

19 Hu, S. C. & Chen,Y. W. *J. Chem. Technol. Biotechnol.* **76**, 954 (2001).

20 Xue, W. *et al.* *Catal. Commun.* **11**, 29 (2009).

21 Hu, S. C. & Chen, Y. W. *Ind Eng Chem Res.* **40**, 6099 (2001).

22 Zhang, P. *et al.* *Green Chem.* **15**, 152 (2013).

23 Fan, G. Y., Li, R. X., Li, X. J. & Chen, H. *Catal. Commun.*, **9**, 1394 (2008).

24 Wang, J. *et al.* *J Mol Catal A: Chem.* **222**, 229 (2004).

25 Ning, J. B., Xu, J., Liu J. & Lu, F. *Catal. Lett.* **109**, 175 (2006).

26 Wang, J. *et al.* *Appl Catal A: Gen*. **272**, 29 (2004).

27 Zhou, G. B. *et al.* *Ind. Eng. Chem. Res.* **51**, 12205 (2012).

28 Zhou, G. B. *et al.* *J. Catal.* **311**, 393 (2014).

29 Liu, H. *et al.* *J Mol Catal A: Chem.* **341**, 35 (2011).

30 Zhou, G. *et al.* *ChemCatChem*, **5**, 2425 (2013).

31 Liu, J. L. *et al.* *Appl Catal A: Gen.*, **353**, 282 (2009).

32 Liu, J. *et al. J. Catal.* **268**, 100 (2009).

33 Liu, Z. Y. *et al.* *Chin. J. Chem*. **28**, 1927 (2010).
